# Non-adherence to diet and exercise recommendations amongst patients with type 2 diabetes mellitus attending Extension II Clinic in Botswana

**DOI:** 10.4102/phcfm.v5i1.457

**Published:** 2013-05-13

**Authors:** Adewale B. Ganiyu, Langalibalele H. Mabuza, Nomsa H. Malete, Indiran Govender, Gboyega A. Ogunbanjo

**Affiliations:** 1 Department of Family Medicine, University of Botswana, Botswana; 2 Department of Family Medicine and Primary Health Care, University of Limpopo, South Africa

## Abstract

**Background:**

Patients diagnosed with type 2 diabetes mellitus in Extension II Clinic in Botswana have difficulty in adhering to the lifestyle modifications recommended by health care practitioners. Poor adherence to lifestyle recommendations leads to poor control of the condition and consequently to complications.

**Objectives:**

The aim of the study was to determine reasons for poor adherence to lifestyle recommendations amongst the patients. The objectives were to determine: reasons for poor adherence to dietary requirements, exercise recommendations, the support they had in adhering to the recommendations, and their understanding of the role of dietary and exercise requirements in the management of their condition.

**Method:**

This was a cross-sectional descriptive study. The sample comprised of 105 participants. Data on participants’ baseline characteristics and adherence to dietary and exercise habits were analysed using the SPSS 14.0 version.

**Results:**

The sample of 104 participants comprised of 61 (58.7%) women. The rates of non-adherence to diet and exercise were 37% and 52% respectively. The main reasons for non-adherence to diet were: poor self-discipline (63.4%); lack of information (33.3%) and the tendency to eat out (31.7%). The main reasons for non-adherence to exercise were: lack of information (65.7%); the perception that exercise exacerbated their illness (57.6%) and lack of an exercise partner (24.0%).

**Conclusion:**

There was a relatively high rate of non-adherence to both diet and exercise recommendations by patients suffering from type 2 diabetes mellitus at Extension II Clinic, Botswana, with non-adherence to exercise recommendations more common.

## Abstrait

**Le non respect des recommandations alimentaires et relatives à l'exercice physique chez les patients souffrant de diabète non insulino-dépendant fréquentant le centre médical d'Extension II au Botswana**

**Contexte:**

Les patients diagnostiqués avec un diabète non insulino-dépendant au centre médical d'Extension II au Botswana ont des difficultés à respecter les modifications que les professionnels de la santé leur recommandent d'apporter à leur style de vie. Le mauvais respect de ces recommandations entraine un mauvais contrôle de leur état de santé et, par conséquent, des complications.

**Objectifs:**

L’étude avait pour but de déterminer les raisons de la faible observance des recommandations relatives au style de vie par les patients. Les objectifs étaient également de déterminer: les raisons de la faible observance des exigences alimentaires, des recommandations relatives à l'exercice physique, le soutien dont ils bénéficiaient pour suivre ces recommandations, et leur compréhension du rôle des exigences alimentaires et relatives à l'exercice physique dans la prise en charge de leur problème de santé.

**Méthode:**

Il s'agissait d'une étude transversale descriptive. L’échantillon se composait de 105 participants. Les données relatives aux caractéristiques de base des participants et au respect des consignes alimentaires et relatives à l'exercice physique ont été analysées en utilisant le logiciel SPSS, version 14.0.

**Résultats:**

L’échantillon de 104 participants se composait de 61 femmes (58,7%). Le taux de non respect des consignes alimentaires et relatives à l'exercice physique était respectivement de 37% et de 52%. Les principales raisons du non respect du régime alimentaire étaient: la difficulté à s'auto-discipliner (63,4%); le manque d'informations (33,3%) et la tendance à manger à l'extérieur (31,7%). Les principales raisons du non respect des consignes relatives à l'exercice physique étaient: le manque d'informations (65,7%); le sentiment que l'exercice aggravait leur état de santé (57,6%) et l'absence de partenaire pour faire de l'exercice (24%).

**Conclusion:**

Le taux de non respect des recommandations alimentaires et relatives à l'exercice physique était relativement élevé chez les patients souffrant de diabète non insulino-dépendant dans le centre médical d'Extension II, au Botswana, le non respect des recommandations relatives à l'exercice étant plus fréquent.

## Introduction

Several studies have shown the benefit of healthy dietary habits and regular exercise in the prevention and management of type 2 diabetes mellitus.^1, 2, 3, 4^ Adherence to prescribed lifestyle changes have also been shown to improve glucose levels, to lead to decreased blood pressure and to correct lipid abnormalities which are factors associated with the micro and macro-vascular complications of diabetes.^5, 6^ Therefore primary prevention based on strict adherence to healthy lifestyle habits must be advocated in health policies worldwide to control diabetes mellitus, particularly in developing countries like Botswana where access to and quality of health care is still under development.^7^

Adherence has been defined as ‘the extent to which a person's behaviour – taking medication, following a diet, and/or executing lifestyle changes - corresponds with agreed recommendations from a health care provider.^8^ Though not perfect, the term ‘adherence’ is preferable to ‘compliance’, since the latter implies patient submission to the health care professional's orders without mutual negotiation.^9^ Studies have been conducted worldwide and in Africa to establish factors associated with non-adherence to treatment amongst patients with type 2 diabetes mellitus.^10, 11^ Nevertheless, there is paucity of studies on compliance to lifestyle recommendations. Amongst factors identified as responsible for poor adherence to the treatment of diabetes mellitus is a poor relationship between the healthcare provider and patient.^12^

Poor adherence to healthy lifestyle recommendations amongst type 2 diabetes mellitus patients has been found to be associated with the global urbanisation of communities (especially developing countries) with an increasing number of fast-food outlets serving unhealthy food.^13^ In these patients, rates of non-adherence to diet and exercise recommendations were estimated to range from 35% – 75% and 35% – 81% respectively in studies conducted outside Africa.^14, 15, 16, 17, 18, 19, 20^ Poor adherence to diet and exercise recommendations in people with type 2 diabetes mellitus is known to manifest itself through frequent hospitalisations leading to increased health care costs. ^21, 22^

Some patients justify their non-adherence to dietary recommendations on the basis of criticism by others, lack of information, unwillingness, lack of support from spouse and/or family, negative health beliefs and perceptions, previous experience with chronic disease and financial problems.^10, 13, 15^ Other common reported barriers for non-adherence to exercise were lack of will-power, poor health, associated co-morbidities, lack of an exercise partner, poor weather (hot and cold conditions) and a busy schedule.^4, 10, 15, 16, 17, 23^

The aim of the study was to determine reasons for poor adherence to lifestyle recommendations in patients with type 2 diabetes mellitus attending the clinic. The objectives were to determine reasons for poor adherence to dietary requirements, reasons for poor adherence to exercise recommendations, who supported them in order to adhere to recommendations, and their understanding of the role of dietary and exercise requirements in the management of their condition.

### Significance of the study

To our knowledge, at the time of the study in 2008, there was a paucity of studies conducted to investigate non-adherence to lifestyle modification recommendations (diet and exercise) amongst type 2 diabetes mellitus patients in Africa and in Botswana in particular. It is hoped that the study will address this gap by establishing the reasons given by the patients for non-adherence to diet and exercise recommendations.

## Ethical considerations

Ethics approval was obtained from the Medunsa Campus Research Ethics Committee (MCREC) of the University of Limpopo in South Africa (ethics clearance number: MCREC/M/12/2008), and the Health Research Unit of the Ministry of Health, Botswana [PPME-13/18/1 PS Vol. III (20)]. Permission for data collection at the study site was also obtained from the District Health Team of the Gaborone City Council, Botswana (GCC/H/8).

## Method

### Setting

From 01 July 2008 to 30 September 2008 a simple descriptive, cross-sectional study was conducted to determine the reasons for non-adherence to diet and exercise recommendations given by health care practitioners to patients with type 2 diabetes at Extension II Clinic, a public health facility in Gaborone, Botswana. This study also elicited patients’ understanding of the role of diet and exercise recommendations in the management of their condition.

### Sampling

The target population were individuals of 30 years and above who had been diagnosed with type 2 diabetes mellitus more than two years previously and had been on treatment at the clinic during these years. This age group was targeted because, according to the clinic records, most of the type 2 diabetes mellitus patients were aged 30 years and above.19 Individuals with type 1 diabetes mellitus, those aged less than 30 years and those who had been diagnosed less than two years before the study commenced were excluded from the study.

### Design and procedure

The clinic for diabetic patients was on Wednesdays and Fridays. Patients seen per month ranged between 38 and 46, accounting for an average of 128 patients per month. Using a confidence level of 95% and 5% confidence interval, the sample size was calculated as 96 participants. For ease of calculation, the sample size was rounded off to 105, that is, 35 participants per month over three consecutive months. Systematic sampling was done with every second patient seen at the diabetic clinic. A total of 105 participants were recruited, comprising of 44 men and 61 women. Informed written consent was obtained from each participant after the objectives of the study had been explained. None of the patients recruited declined to be part of the study. Anonymity and confidentiality of data were assured by non-inclusion of patient identifiers in the questionnaires. The research team was guided by a literature search in the formulation of the questions relevant for the study.^11, 21^ Each consenting patient was requested to fill in the structured questionnaire with the help of the research team members who were on hand to offer clarity where necessary. In our study, a respondent was regarded adherent to exercise if she or he reported exercising for a duration of ≥ 30 minutes per session, most days of the week.^24^ We defined non-adherence to exercise as a self-reported default for more than three days per week.^25^ Dietary recommendations comprised of a recommendation by a health care professional of a Dietary Approach to Stop Hypertension (DASH) diet comprising of whole grains and fibre (more than 5 portions), fruits and vegetables (at least 2 servings of each), lean meats, poultry and fish (at most 3 servings), low-fat milk and dairy products (at most 3 servings) and small amounts of fats, oils, refined sugars and salt.^26, 27, 28^ We defined non-adherence to dietary recommendations as self-reported adherence of less than three days a week (seldom).^29^ However, the researchers noted that the World Health Organization (WHO) Adherence Project indicates that regarding adherence measurement, ‘no single measurement strategy has been deemed optimal. A multi-method approach that combines feasible self-reporting and reasonable objective measures is the current state-of-the-art in measurement of adherence behaviour.^30^

Data was collected from 01 July 2008 to 30 September 2008 using a questionnaire in English and Setswana (local language) and using the Microsoft Excel^®^ software programme and subsequently exported to the SPSS 14.0 version for analysis.

## Results

One hundred and five questionnaires were distributed to consenting participants; one had missing data on most sections and was discarded. A response rate of 100% was obtained.

### Baseline characteristics of participants

Participants’ baseline characteristics are tabulated (Table 1), of the 104 participants, 61 (58.7%) were females. A third of the participants, 28 (26.9%) aged 50 to 59 years. The 30 to 39 year age group was the least represented, 15 (14.4%) participants. Above eighty percent (83.6%) of the participants had formal education while 16.4% had none.

**TABLE 1:** Distribution of participants’ baseline characteristic.

Characteristic	Variable	*n*	%
**Gender**	Male	43	41.3
	Female	61	58.7
**Age (years)**	30–39	15	14.4
	40–49	22	21.2
	50–59	28	26.9
	60–69	20	19.2
	70 and above	19	18.3
**Marital status**	Single	27	26.0
	Married	43	41.3
	Divorced	05	4.8
	Separated	03	2.9
	Co-habiting	15	14.4
	Widowed	11	10.6
**Educational level**	None	17	16.4
	Primary	23	22.1
	Secondary	34	32.7
	Tertiary	30	28.8
**Employment status**	Unemployed	16	15.4
	Employed	57	54.8
	Pensioner	19	18.3
	Housewife	12	11.5

*n*, sample size;%, percentage.

#### Reasons for non-adherence to lifestyle modifications

Table 2 shows that more than one third, 38 participants (37%; 95% CI, 27.7–46.3) and slightly over half, 54 participants(52%; 5% CI, 42.4–61.6) did not adhere to diet and exercise recommendations, respectively.

**TABLE 2:** Non-adherence to lifestyle modifications and reasons given.

Non-adherance	Barriers	Reasons	%	CI
Diet			**37.0**	**27.7–46.3**
	Barriers to diet recommendations	Eating out	31.7	23.0–41.0
		Financial constraints	28.8	20.3–37.7
		Poor self-discipline	63.4	53.6–72.2
		Eating at another's home	18.3	10.6–25.4
		Situation at home	6.7	2.1–11.9
		Lack of information	33.3	24.0–42.2
Exercise	Barriers to exercise recommendations		**52.0**	**42.4–61.6**
		Weather	15.4	8.1–21.8
		Lack of exercise partner	24.0	15.8–32.2
		Specific location	18.0	10.6–25.4
		Criticism	1.9	-0.7–4.7
		Lack of in formation	65.7	56.1–75.0
		Exercise exacerbating illness	57.6	48.5–67.5
	Emotional support	Spouse	54.1	44.4–63.4
		Family members	44.8	35.4–54.6
		Friends	58.7	49.6–68.5

%, percentage; CI, 95% Confidence Interval.

#### Reasons for non-adherence to dietary recommendations

Reasons given for non-adherence to diet recommendations were poor self-discipline (63.4%; 95% CI, 53.6–72.2), lack of information on a healthy diet (33.3%; 95% CI, 24.0–42.2), eating out, e.g. in restaurants (31.7%; 95% CI, 23.03–40.97) and financial constraints in accessing the diet recommended by health care practitioners (28.8%; 95% CI, 20.3–37.7). The reason least mentioned was their home situation – ingesting unhealthy diets when alone (6.7%; 95% CI, 2.1–11.9). (see Table 2)

#### Reasons for non-adherence to exercise recommendations

Fifty-two percent did not exercise regularly, because of a lack of information about the benefit of exercise and how it should be done (65.7%; 95% CI, 56.1–75.0), the notion that exercise exacerbated diabetes mellitus (57.6%; 95% CI, 48.5–67.5), lack of an exercise partner (24%; 95% CI, 15.8–32.2), going from home, e.g. to cattle posts, on official trips, or to other areas (18%; 95% CI, 10.6–25.4), and extreme weather conditions (very cold winters and very hot summers) (15.4%; 95% CI, 8.1–21.8). The least mentioned reason for not adhering to exercise was criticism by others (friends and family members) (1.9%; 95% CI, -0.69–4.69), with the confidence interval crossing the line of no difference (1.00).

**FIGURE 1:**
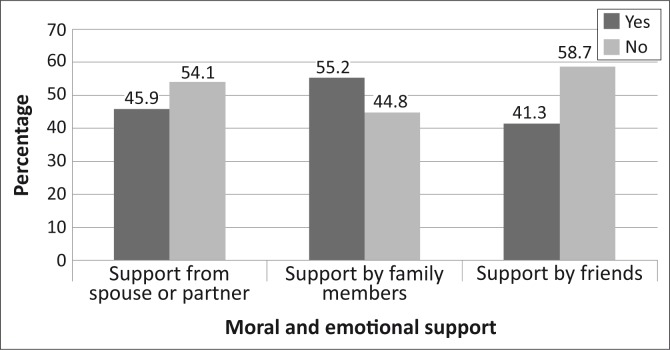
Bar chart demonstrating moral and emotional support from spouse or partner, family members and friends.

Figure 1 illustrates that poor emotional support from a spouse/partner (54.1%; 95% CI, 44.4–63.4) and friends (58.7%; 95% CI, 49.6–68.5) contributed to non-adherence to diet and exercise recommendations. However, other family members (55.2%; 95% CI, 42.5–61.7) were reported to be supportive.

In Table 3 it can be seen that 48.1% of the participants understood recommendations regarding lifestyle measures as referring to both healthy dietary habits and exercise, while 36.5% and 15.4% respectively understood lifestyle modifications as referring exclusively to healthy dietary habits or exercise. Almost all (95.1%) understood that healthy dietary habits help to control blood sugar levels. Similarly, slightly over two-thirds (67.3%) claimed they understood that exercise helped to control diabetes mellitus.

**TABLE 3:** Patients’ understanding of diet and exercise recommendations.

Patients understanding	Variable	*n*	%
Life style recommendations	Diet only	38	36.5
	Exercise only	16	15.4
	Both diet and exercise	50	48.1
Diet helps to control blood sugar level	Yes	99	95.1
	No	05	4.9
Exercise helps to control blood sugar	Yes	70	67.3
	No	34	32.7

*n*, sample size;%, percentage.

## Discussion

Targeting lifestyle modifications amongst patients with type 2 diabetes mellitus is effective if the healthcare practitioner understands patients’ reasons for adherence and non-adherence to diets and exercise recommendations. Certain studies indicate that adherence to prescribed diet and regular exercise are important for both prevention and control of patients with type 2 diabetes mellitus.^3, 31, 32^ Our study demonstrated trends and patterns in the patients’ responses to reasons for non-adherence to recommendations on lifestyle modifications relating to diet and exercise. Understanding the reasons for non-adherence can facilitate intervention strategies.

### Prevalence of non-adherence to lifestyle recommendations

This study showed that more than one-third (37.2%) and nearly half (52.0%) of the participants did not adhere to diet and exercise recommendations respectively, but non-adherence to exercise was commoner than non-adherence to diet. We hypothesise that reasons for the difference in the rates might be related to differences in patients’ understanding and perceptions of the role of diet and exercise in the control of diabetes mellitus. More than half (57.6%) of non-adherent patients thought that exercise would exacerbate their illness, one of the reasons being that they experienced body pains during and after exercising.

The rates of non-adherence to diet and exercise in this study compared well with those reported in previous studies.^17, 18, 19, 20, 21^ However, non-adherence to diet and exercise from this study (37.4% and 52%, respectively) appeared to be slightly lower than those reported in other countries where similar studies were conducted (> 40% and > 55% for non-adherence to diet and exercise, respectively).^15, 19, 20, 22, 23^ This may be ascribed to the smaller sample size used in this study compared to those studies that reported higher rates of non-adherence.

### Reasons for non-adherence to both diet and exercise recommendations

Despite the fact that most participants understood that diet and exercise were important to achieve and maintain good glycaemic control, the majority still gave various reasons for their non-adherence to these recommendations. The most frequently reported reasons for non-adherence to dietary recommendations were poor self-discipline, lack of information, eating out (especially at fast-food outlets, social gatherings, and the homes of extended families and friends) and financial constraints.

On the other hand, the reasons given for non-adherence to exercise recommendations were lack of information on the benefits of exercise, the view that exercise worsened their condition, lack of an exercise partner, being away from home (e.g. at social gatherings, on official trips and at cattle posts), and extreme weather conditions (very cold winters and very hot summers). These findings are consistent with the observations noted in previous studies on nutrition and adherence to an exercise regimen conducted in the first world (USA) as well as in developing countries. The populations studied consisted of patients with type 2 diabetes mellitus who were discovered not to engage in recommended levels of physical activity and dietary guidelines for *inter alia* fruit and vegetable consumption.^15, 20, 23^

Lack of emotional support from the spouse and friends was claimed to have contributed to non-adherence to diet and exercise recommendations. Other studies found that good support from spouse, family members and friends were good predictors to adherence to diet and exercise recommendations.^19, 20, 21, 31^ Our study demonstrated that, although there was a lack of support from friends and the spouse/partner of a patient with type 2 diabetes mellitus, there was reasonable support from other family members (55.2%). This finding is supported by the fact that there is strong family cohesion and support amongst traditional societies such as those found in Botswana.^21^ Adherence to diet does require strong support from the patient's family, as meals are usually shared by all members in a family. In the study setting, this finding should be factored in during diabetes education.

In this study, lack of information (including written instruction) from health care providers appeared to be the most frequently reported reason for non-adherence to diet and exercise recommendations. This barrier was more common in exercise non-adherence (65.7%); it was approximately double the finding for diet non-adherence (33.3%). This finding supports similar studies undertaken on the subject of lifestyle modification adherence.^20, 31, 33^ It is the responsibility of the health care provider to provide adequate information on diet and exercise regimens to the patient as part of a holistic health care package. Patients with diabetes may not strictly adhere to lifestyle measures unless they are educated. Individualised lifestyle measures may be achieved through ‘assessment of the patient's knowledge and needs, anticipation of the individual's future barriers’ and identification of their support structures.^33^

Our study demonstrated that eating away from home resulted in non-adherence to diet and exercise recommendations. This finding is consistent with cultural norms in the Republic of Botswana, where an individual has more than one home, such as a city home, a village home and a home at the cattle post. The individual's dietary and exercise habits differ in each location. Therefore it is important to assess and address the influence of alternative homes on adherence to diet and exercise regimens during diabetic education. A similar observation was made in a study that demonstrated that another person's home (14%) and specific locations away from home (20%) were associated with non-adherence to diet and exercise recommendations.^20^

### Patients’ understanding of lifestyle modification recommendations

This study established that most of the participants’ understanding of diet and exercise had a direct influence on their adherence to diet and exercise recommendations. One in two respondents had a general understanding that diet and exercise were important lifestyle measures by which to improve their diabetic control. This finding was consistent with studies done elsewhere that reported that individuals with type 2 diabetes felt that diet and exercise could have a positive effect on their glycaemic control.^15, 23^ In our study, one explanation for this understanding may be that most participants had a relatively high level of formal education (83.6%). Understanding cannot be equated to patient practice, however, a study in Uganda also found no significant association between the level of education and the management of diabetes mellitus.^11^ This finding on patient understanding should be seen as an advantage by a health care provider and factored in when planning diet and exercise regimens for diabetes education amongst patients. Patient education on medication, diet and exercise were shown to significantly improve glycaemic control and health-related quality of life in a clinical trial conducted over a twelve-month period amongst type 2 diabetes patients attending a military hospital outpatient clinic in the United Arab Emirates.^34^

### Strengths and limitations of the study

This study investigated a subject not previously explored where there was a paucity of data. It reported reasons given by patients with type 2 diabetes mellitus in Gaborone, Botswana, for non-adherence to lifestyle modifications (diet and exercise). The study was conducted in a primary care setting, which makes it more relevant to the primary care practitioner. The study did not elicit whether there was prior instruction to the patients with type 2 diabetes mellitus on exercise and diet recommendations. It was based on the assumption that such instruction is given to patients during diabetes education. There was a limitation in the sampling method as the researchers excluded patients diagnosed less than two years before the study was conducted. The use of a structured questionnaire also limited the responses to the questions posed, as it excluded other possible reasons for non-adherence to lifestyle recommendations. The sample size was time-bound (over three months) due to financial constraints. The setting was limited to only one primary care centre out of 15 primary care centres in Gaborone, which may affect generalisability. A nationwide study covering all or most of the 15 primary care centres in Gaborone should shed more light in the subject.

### Recommendations

There is a need for patient education and health promotion to address the lack of information on a healthy diet as well as the lack of information on the benefits of exercise and how exercise should be undertaken. There is also a need to investigate and address the notion by patients that exercise exacerbates diabetes mellitus.

## Conclusion

There was a high rate of non-adherence to diet and exercise recommendations by patients suffering from type 2 diabetes mellitus seen at Extension II Clinic, Gaborone, Botswana. Non-adherence to exercise recommendations was more common than non-adherence to diet. The most common reasons for non-adherence to diet were poor self-discipline, lack of information, eating out and financial constraints. Lack of information, the perception that exercise exacerbates the illness, lack of an exercise partner, adverse weather and locations away from home were the most frequently reported reasons for not adhering to exercise recommendations. A lack of emotional support from the spouse, friends and to a lesser extent family members were the reported contributing factors for non-adherence to diet and exercise recommendations.
